# Medical therapy of MASLD – consequences of the paradigm shift from histology to non-invasive tests

**DOI:** 10.1016/j.jhepr.2026.101996

**Published:** 2026-09-03

**Authors:** Eva Katharina Messer, David Petroff, Katsutoshi Sugimoto, Philip N. Newsome, Fuminori Moriyasu, Shalimar, Marco Dioguardi Burgio, Cosmin Sebastian Voican, Valérie Vilgrain, Victor de Lédinghen, Daniel Jesper, Stephan Baumeler, Deike Strobel, Wah Kheong Chan, Edward G. Grant, Gabriel Perlemuter, Linda C. Kelahan, Ana-Carolina Cardoso, Helena Gabriel, Sandeep Aggarwal, Byung Ihn Choi, Peter J. Eddowes, Takashi Nishimura, Michael Allison, Hiroko Iijima, Emmanuel Tsochatzis, Theodore J. Dubinsky, Quentin M. Anstee, Jing Gao, David Sheridan, Dong Ho Lee, Jeremy F. Cobbold, Jae Young Lee, Sylvie Naveau, Yanan Zhao, David Semela, Pintong Huang, Grace Lai-Hung Wong, Jie Zeng, Vincent Wai-Sun Wong, Adrian Lim, Cristiane A. Villela-Nogueira, Xiaoyan Xie, Harshit Garg, Richard G. Barr, Vito Cantisani, Giovanna Ferraioli, Kentaro Sakamaki, Takao Itoi, Masayoshi Kage, Hirohisa Yano, Valentin Blank, Johannes Wiegand, Thomas Karlas

**Affiliations:** 1 Division of Hepatology, Department of Medicine II, Leipzig University Medical Centre, Leipzig, Germany; 2 LeiCeM - Leipzig Center of Metabolism, Leipzig University, Leipzig, Germany; 3 Clinical Trial Centre Leipzig, Leipzig University, Leipzig, Germany; 4 Department of Gastroenterology and Hepatology, Dokkyo Medical University Saitama Medical Center, Saitama, Japan; 5 Roger Williams Institute of Liver Studies, Faculty of Life Sciences and Medicine, King’s College London, Foundation for Liver Research & King’s College Hospital, London, United Kingdom; 6 College of Medical and Dental Sciences, University of Birmingham, Birmingham, United Kingdom; 7 Department of Gastroenterology and Hepatology, Tokyo Medical University, Tokyo, Japan; 8 Department of Gastroenterology, All India Institute of Medical Sciences, New Delhi, India; 9 Université Paris Cité, INSERM, Centre de recherche sur l’inflammation, Paris, France; 10 Department of Radiology, Hôpital Beaujon, AP-HP Nord, Clichy, France; 11 Faculté de Médecine Paris-Sud, Université Paris-Sud, Université Paris-Saclay, Le Kremlin-Bicêtre, France; 12 Service d’Hépato-Gastroentérologie et Nutrition, Hôpital Antoine-Béclère, Hôpitaux Universitaires Paris-Sud, Assistance Publique-Hôpitaux de Paris, Clamart, France; 13 INSERM U996, DHU Hepatinov, Labex LERMIT, Clamart, France; 14 Centre d’Investigation de la Fibrose Hépatique, Bordeaux University Hospital, Pessac, France; 15 INSERM U1053, Bordeaux University, Bordeaux, France; 16 Departments of Internal Medicine 1, Medicine 1, Gastroenterology, Endocrinology, and Pneumology, Erlangen University Hospital, Erlangen, Germany; 17 Department of Gastroenterology and Hepatology, Cantonal Hospital Lucerne, Lucerne, Switzerland; 18 Gastroenterology and Hepatology Unit, Department of Medicine, Faculty of Medicine, University of Malaya, Kuala Lumpur, Malaysia; 19 Department of Radiology, Keck Hospital of USC, University of Southern California, Los Angeles, California, USA; 20 Department of Radiology, Northwestern Memorial Hospital, Northwestern University Feinberg School of Medicine, Chicago, Illinois, USA; 21 Hepatology Unit, Hospital Universitário Clementino Fraga Filho, Federal University of Rio de Janeiro, Rio de Janeiro, Brazil; 22 Department of Surgical Disciplines, All India Institute of Medical Sciences, New Delhi, India; 23 Department of Radiology, Chung-Ang University Hospital, Seoul, South Korea; 24 National Institute for Health Research, Birmingham Biomedical Research Centre, University Hospitals Birmingham NHS Foundation Trust, University of Birmingham, Birmingham, United Kingdom; 25 Centre for Liver Research, Institute of Immunology and Immunotherapy, University of Birmingham, Birmingham, United Kingdom; 26 Liver Unit, University Hospitals Birmingham NHS Foundation Trust, Birmingham, United Kingdom; 27 National Institute for Health Research Nottingham Biomedical Research Centre, Nottingham University Hospitals NHS Trust, Nottingham, United Kingdom; 28 University of Nottingham, Nottingham, United Kingdom; 29 Division of Gastroenterology and Hepatology, Department of Internal Medicine, Hyogo College of Medicine, Nishinomiya, Japan; 30 Cambridge Liver Unit, Cambridge NIHR Biomedical Research Centre, Cambridge, United Kingdom; 31 University College London Institute for Liver and Digestive Health, Royal Free Hospital, London, United Kingdom; 32 Department of Radiology, University of Washington, Seattle, Washington, USA; 33 Translational and Clinical Research Institute, Faculty of Medical Sciences, Newcastle University, Newcastle upon Tyne, United Kingdom; 34 Newcastle National Institute for Health Research Biomedical Research Centre, Newcastle upon Tyne Hospitals NHS Foundation Trust, Newcastle upon Tyne, United Kingdom; 35 Rocky Vista University, Ivins, Utah, USA; 36 Institute of Translational and Stratified Medicine, Faculty of Medicine and Dentistry, University of Plymouth, Plymouth, United Kingdom; 37 Department of Radiology, Seoul National University Hospital, Seoul, South Korea; 38 Department of Gastroenterology and Hepatology, Oxford University Hospitals NHS Foundation Trust, John Radcliffe Hospital, Oxford, United Kingdom; 39 Department of Ultrasound in Medicine, Second Affiliated Hospital, Zhejiang University School of Medicine, Hangzhou, China; 40 Department of Gastroenterology and Hepatology, Cantonal Hospital St. Gallen, St. Gallen, Switzerland; 41 Institute of Digestive Disease, The Chinese University of Hong Kong, Sha Tin, Hong Kong, China; 42 Medical Data Analytics Centre (MDAC), Department of Medicine and Therapeutics, The Chinese University of Hong Kong, Sha Tin, Hong Kong, China; 43 Department of Ultrasound, Third Affiliated Hospital of Sun Yat-sen University, Guangzhou, China; 44 Department of Imaging, Charing Cross Hospital, Imperial College London and Imperial College Healthcare NHS Trust, London, United Kingdom; 45 Department of Medical Ultrasonics, Institute of Diagnostic and Interventional Ultrasound, First Affiliated Hospital of Sun Yat-sen University, Guangzhou, China; 46 Department of Radiology, Northeastern Ohio Medical University, Rootstown, Ohio, USA; 47 Division of Internal-Surgical Ultrasound, Department of Radiologic, Oncologic, and Anatomic-Pathologic Science, Polyclinic Umberto I, Sapienza University of Rome, Rome, Italy; 48 Department of Clinical, Surgical, Diagnostic, and Pediatric Sciences, Medical School, University of Pavia, Pavia, Italy; 49 Centre for Data Science, Juntendo University, Tokyo, Japan; 50 Department of Pathology, Kurume University School of Medicine, Kurume, Japan; 51 Division of Gastroenterology, Department of Medicine II, Leipzig University Medical Centre, Leipzig, Germany; 52 Division of diagnostic and interventional ultrasound, Department of Internal Medicine I, University Hospital Halle, Halle, Germany

**Keywords:** Metabolic Dysfunction-Associated Steatohepatitis, LeiCeM, Multiparametric Ultrasound, Transient Elastography, Controlled Attenuation Parameter, Dispersion Slope, Attenuation Coefficient, Shear-Wave Speed, Liver Stiffness

## Abstract

**Background & Aims:**

The recent approval of pharmacological therapies for fibrotic metabolic dysfunction-associated steatohepatitis (MASH) has increased the need for accurate identification of treatment-eligible patients. Current recommendations increasingly rely on non-invasive tests (NITs), including vibration-controlled transient elastography (VCTE), while multiparametric ultrasound (MPUS) may provide additional opportunities for non-invasive assessment. However, agreement between histology and imaging-based approaches remains uncertain. We compared treatment eligibility based on histology, VCTE, and MPUS in two international biopsy-proven cohorts of metabolic dysfunction-associated steatotic liver disease (MASLD).

**Methods:**

We analysed two biopsy-proven MASLD cohorts: CAP-IPDMA (n = 1,029), including VCTE and controlled attenuation parameter (CAP), and iLEAD (n = 124), including MPUS with two-dimensional shear wave elastography (2D-SWE). Treatment eligibility was assessed using histologically confirmed F2/F3 MASH and NIT-based recommendations from international expert panels.

**Results:**

In CAP-IPDMA, 277/1,029 patients (26.9%) met the histological definition of “at-risk MASH”. Depending on the VCTE cut-off, 13.2-32.0% qualified for treatment. Overlap between histological “at-risk MASH” and VCTE thresholds was limited, reaching 27.8% when using VCTE 8-15 kPa, and decreasing when narrower or higher thresholds were applied. Among patients identified only by VCTE 8-15 kPa, males had lower median AST and ALT than those fulfilling only the histological indication (35 *vs*. 48 IU/L *p* = 0.034 and 45 *vs*. 62 IU/L *p* = 0.0072, respectively). In iLEAD, 21/124 patients (16.9%) met the histological definition, while 13.7–16.1% were eligible based on 2D-SWE thresholds, again with similarly limited overlap.

**Conclusions:**

Histology and NITs capture partly distinct patient populations, meaning that both the number and type of patients selected for therapy depend on the chosen modality and cut-offs. As VCTE and MPUS become increasingly accessible in clinical practice, prospective validation is essential for establishing reliable non-invasive treatment pathways.

**Impact and implications:**

The current literature reflects a paradigm shift away from biopsy-based approaches toward non-invasive test (NIT)-based assessment of treatment eligibility in metabolic dysfunction–associated steatotic liver disease (MASLD), which may substantially affect which patients receive newly approved therapies. Our results are important for clinicians, researchers, and guideline developers because histology and current NIT cut-offs identify only partially overlapping patient populations, implying that different diagnostic strategies select different risk profiles. In practice, these findings support thoughtful implementation of NIT-based treatment pathways, the use of repeated measurements, and prospective validation of NIT thresholds to guide clinical care, trial design, and health policy decisions.

## Highlights

• Histology and NITs identify largely different populations with at-risk MASH.
• Choice of VCTE cut-offs strongly impacts the proportion of patients selected.
• FAST score enriched for histologically defined MASH.
• Prospective validation of NIT-based treatment pathways is urgently needed.

## Graphical abstract

**Figure:**
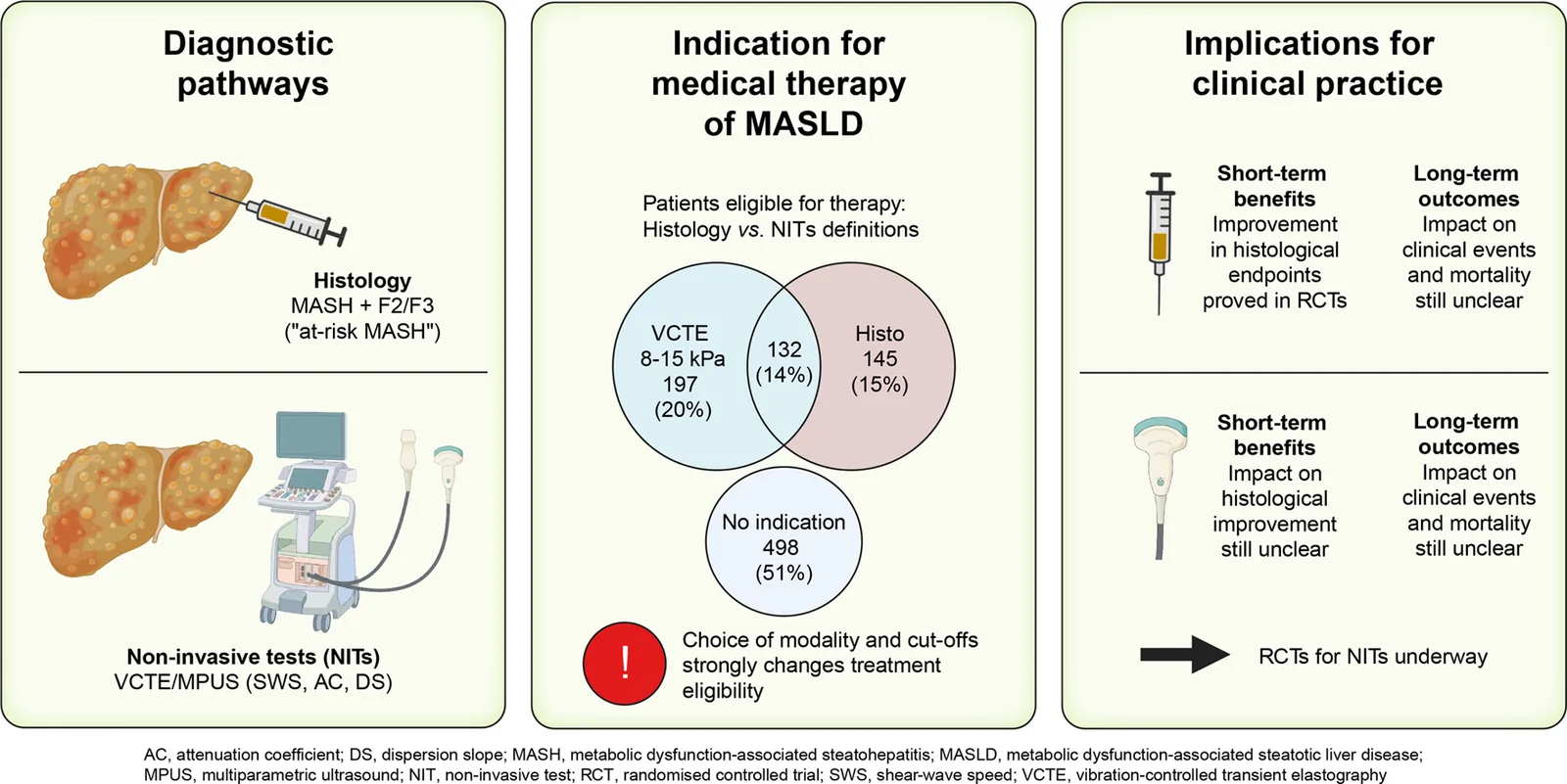

## Introduction

Metabolic dysfunction-associated steatotic liver disease (MASLD) is expected to affect over half of the global adult population by 2040.[1], [2] This will be accompanied by an increase in the prevalence of metabolic dysfunction-associated steatohepatitis (MASH) to over 20%, with a rise in the subsequent risk of liver-related complications, including hepatocellular carcinoma (HCC), hepatic decompensation, and mortality.[2], [3], [4] Currently, two pharmacological treatment options are available for patients with MASH and fibrosis stages F2–F3 (“at-risk MASH”). Resmetirom, a liver-directed, β-selective thyroid hormone receptor agonist, was the first drug to receive conditional approval, followed by semaglutide, a glucagon-like peptide-1 receptor agonist.[5], [6], [7], [8] These regulatory decisions were based on pivotal phase III trials demonstrating significant resolution of steatohepatitis and reduction in fibrosis. Both trials enrolled patients with histologically confirmed fibrotic MASH, defined by a NAFLD activity score ≥4 and fibrosis stages F1–F3 for resmetirom and F2–F3 for semaglutide, and defined treatment success based on histological improvement.[9], [10]

Given the overwhelming prevalence of MASLD and the high number of individuals at risk for MASH, efficient and reliable patient selection for specialized treatment is essential to treat appropriate cases and not to overstrain health care and health insurance systems.^11^

A paradigm shift is therefore underway, moving from liver biopsy as the traditional gold standard for risk assessment to the broader use of non-invasive tests (NITs). This transition is driven by procedural limitations, poor feasibility, and potential procedure-associated complications of liver biopsy.^12^ Consequently, histologic assessment of “at-risk MASH” is not explicitly required by drug labels,[6], [13], [14] underscoring the need for NITs.

Ultrasound-based techniques have shown promise for non-invasive MASH assessment. One established modality is vibration-controlled transient elastography (VCTE) for determining hepatic fibrosis,[15], [16], [17] combined with the controlled attenuation parameter (CAP) for estimating hepatic fat content.[18], [19], [20] These methods require dedicated instruments, whereas two-dimensional shear wave elastography (2D-SWE) is an important alternative that can be implemented in standard ultrasound devices, allowing simultaneous structural imaging and liver stiffness measurement.[20], [21] A recent prospective multicentre study examined the associations between multiparametric ultrasound (MPUS, including 2D-SWE) and histological features, which suggested a broader role for 2D-SWE in non-invasive MASLD evaluation.^22^ Beyond stiffness-based assessment, MPUS also provides quantitative parameters related to hepatic fat content and tissue viscosity/inflammatory activity, which may be useful for future non-invasive treatment stratification and monitoring in MASLD.

Following the approval of pharmacological therapies, several expert opinions have addressed how to assess “at-risk MASH” with VCTE in lieu of a liver biopsy. American Expert Panel Recommendations, also adopted by other societies, aim for MASLD patients without thrombocytopenia and evidence of portal hypertension combined with either VCTE 10–20 kPa (10-15 kPa definitive treatment recommendation, 15–20 kPa consideration for treatment) or FAST (Fibroscan + aspartate aminotransferase [AST]) score ≥0.67.^23^ The AASLD Practice Guidance for MASLD broadens the VCTE interval to 8–15 kPa.^24^ Patients with advanced liver fibrosis (F3/F4) can be identified using VCTE >12 kPa.^12^ However, all of these recommendations do not yet take 2D-SWE into account and were established in different clinical contexts.

The clinical consequences of the differences between treatment indications based on histological and NIT definitions raise questions.[25], [26] With access to more than 1,000 MASLD datasets employing histology and VCTE as well as 124 datasets employing histology and 2D-SWE, we set out to analyze treatment indications based on histology and NITs in a large and multi-national group of patients.[20], [22] In the iLEAD cohort, we additionally explored whether further MPUS parameters beyond 2D-SWE provide complementary information in this context.

## Patients and methods

### Patients and study design

The present analysis is based on the aforementioned existing cohort datasets, referred to below as “CAP-IPDMA”^20^ and the “iLEAD” cohort,^22^ the former of which was registered in the PROSPERO database (CRD42018099284) and the iLEAD cohort in ClinicalTrials.gov (NCT04012242).

Both cohorts included patients with biopsy-confirmed MASLD. CAP-IPDMA represents a meta-analysis-derived patient cohort. For the present analysis, the inclusion criteria led to the use of CAP-IPDMA data from nine original papers, with participating centres in Brazil, France, Hong Kong, India, Malaysia, Switzerland and the United Kingdom. The CAP-IPDMA dataset provides measurements obtained via VCTE and CAP collected between 2013 and 2018, along with the FAST score.

The iLEAD cohort includes data from MPUS assessments collected between 2019 and 2023, including the dispersion slope (DS), attenuation coefficient (AC), and 2D-SWE assessing shear-wave speed (SWS). Overall, nine centres took part in the original study, these were located in Japan, France, Germany, USA, South Korea, Italy, the United Kingdom and China.

The individual case inclusion criteria were as follows:

- Histologically confirmed diagnosis of MASLD/MASH
- Availability of grading and staging based on the Nonalcoholic Steatohepatitis Clinical Research Network (NASH CRN) system; steatosis grade ≥S1
- Availability of the NAFLD activity score (NAS) and fibrosis stage
- Complete elastography data, including liver stiffness measurement via VCTE or SWS, and liver fat assessment using either CAP or attenuation imaging
- VCTE measurements with technical validation and reliability according to the criteria defined by Boursier *et al.*, 2013;^16^ SWS and attenuation imaging assessments in compliance with the iLEAD study protocol^22^
- Alcohol consumption <30 g/d (male) or 20 g/d (female)

The indications for pharmacological therapy were evaluated using multiple approaches:

- Histology: The inclusion criteria of the pivotal phase III trials were used, in alignment with the current FDA labels, which define eligibility as the presence of MASH with fibrosis stages F2 or F3 (“at-risk MASH”).[9], [10], [13], [14]
- CAP-IPDMA cohort: Several non-invasive criteria were applied based on established guidelines:
  ○ American Expert Panel Recommendations:^23^
    ▪ Platelet count ≥140 × 10^9^/L and no evidence of portal hypertension, combined with one of the following:
• VCTE 10–15 kPa (definitive treatment recommendation)
• VCTE 15–20 kPa (consideration for treatment)
• FAST score ≥0.67^27^
  ○ AASLD Practice Guidance for MASLD:^24^
    ▪ VCTE 8–15 kPa
  ○ Advanced fibrosis (F3/F4):^12^
    ▪ VCTE 12-20 kPa

iLEAD cohort: The potential to determine eligibility for pharmacological treatment was primarily assessed using SWS as the stiffness-based MPUS parameter. For better comparability and interpretation of liver stiffness, the “Rule of Four” (5 – 9 – 13 – 17 kPa, as proposed by the Society of Radiologists in the Ultrasound consensus update to identify compensated advanced chronic liver disease (cACLD)^28^) was applied and chosen as follows:

○ 1.7-2.1 m/s (≈9-13 kPa) (suggestive of cACLD)
○ 1.7-2.3 m/s (≈9-16 kPa) (high probability of cACLD).

Further quantitative MPUS parameters, including AC and DS, were analysed to assess whether they may provide complementary information for future non-invasive treatment stratification.

### Statistical analysis

The “American Expert Panel Recommendations” quoted above require a platelet count ≥140 × 10^9^/L to exclude patients at high risk of cirrhosis. When performing analyses that make use of this definition and when comparing it with other definitions, patients with low platelet counts were excluded.

Primarily descriptive statistics were used with the summary statistics mean ± standard deviation, frequencies and median [interquartile range]. When comparing groups formally, we use a *t*-test if data are sufficiently normally distributed, the Wilcoxon-Mann-Whitney test for highly skewed data or a chi-squared test without Yates’ correction for count data. Venn diagrams were created with the “eulerr” package, where the surface areas scale with the proportion of elements.^29^ Because the thrust of the paper puts different diagnostic approaches on equal footing, we do not perform receiver-operating characteristic analyses, which would require a gold standard.

All analyses were performed with the software R 4.5.1.^30^

### Ethics statement

Primary projects already anticipate further use of the data. For the iLEAD cohort, an international vote from the Japanese study centre is available and has been confirmed by the local ethics committees (Leipzig ethics vote 525/25-ek). The analysis of the CAP-IPDMA cohort data was centrally approved by the local ethics committee (ethics vote 218/15-ek) and by each individual study. Informed consent was obtained from all individuals.

Please see the Supplementary CTAT Table for additional study resource information.

## Results

### Patients

From the CAP-IPDMA cohort, 1,029 patients with MASLD and complete histological data and VCTE were analyzed, see Fig. 1 for a flowchart of patient selection. All 124 patients without steatosis grade S0 from the iLEAD cohort were analyzed.

**Fig. 1:**
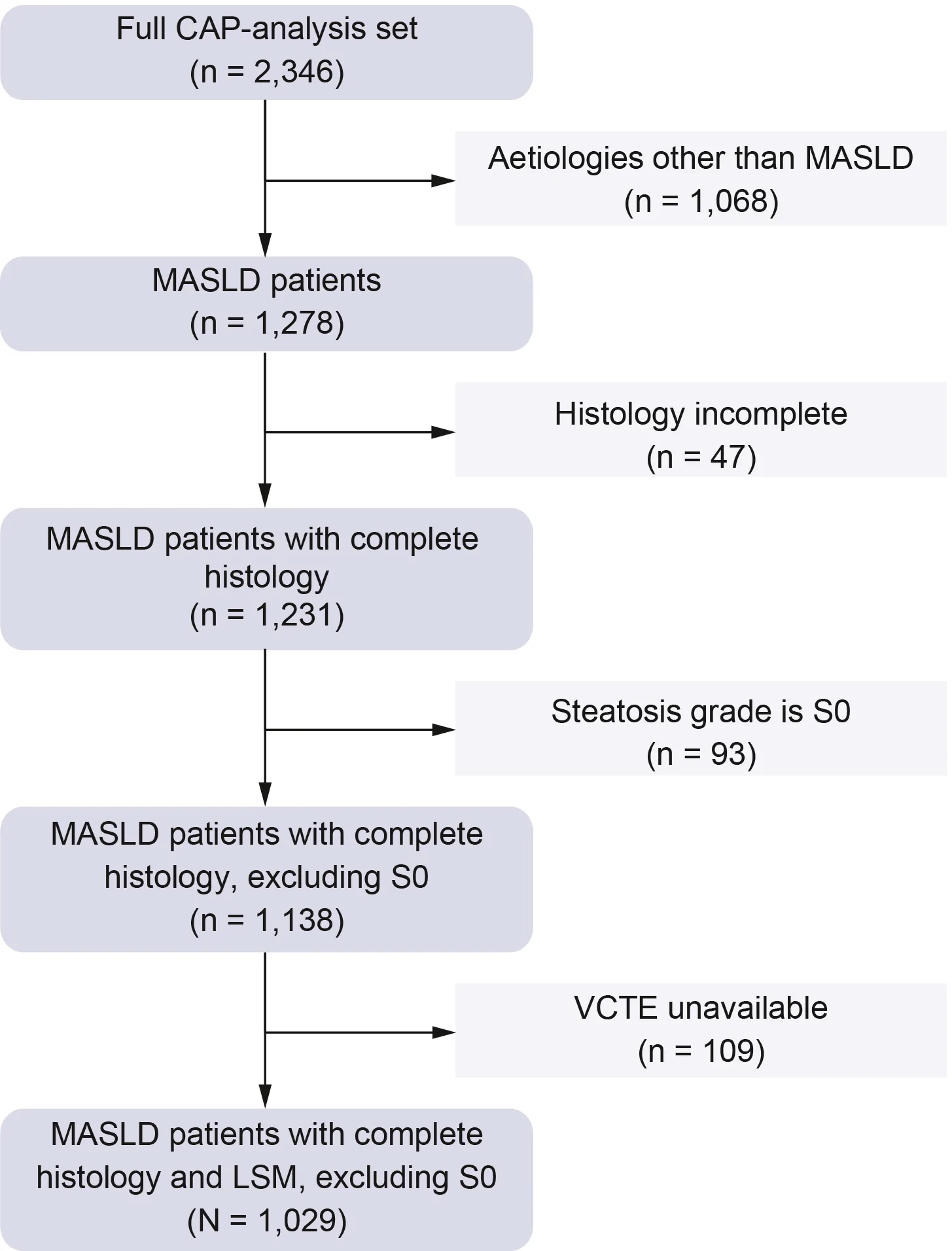
Patient selection from the CAP-IPDMA cohort. CAP, controlled attenuation parameter; LSM, liver stiffness measurement; MASLD, metabolic dysfunction-associated steatotic liver disease; VCTE, vibration-controlled transient elastography.

The patient characteristics of both cohorts are presented in Table 1. The cohorts were fairly similar in terms of age, sex, and proportion of patients with the indications for medical therapy (29% for CAP-IPDMA and 23% for iLEAD). However, whereas the breakdown of F0 and F1 is 19.0% and 39.0%, respectively, for CAP-IPDMA, it is 37.1% and 25.0% for iLEAD. Moreover, aminotransferase levels were higher in the iLEAD cohort. In the iLEAD cohort, ultrasound examinations were performed on the same day as the liver biopsy, and in the CAP-IPDMA cohort, the longest elapsed time was 183 days and was longer than 14 days for only 58 patients (5.6%). The biopsy and ultrasound were performed on the same day in 587 (57.0%) patients.

**Table 1:** Patient characteristics.

	CAP-IPDMA			iLEAD		
	All (N = 1,029)	Histological indication for therapy (n = 297)	No histological indication for therapy (n = 732)	All (n = 124)	Histological indication for therapy (n = 29)	No histological indication for therapy (n = 95)
Number of females	571 (55.5%)	144 (48.5%)	427 (58.3%)	62 (50.0%)	15 (51.7%)	47 (49.5%)
Age (years)	49.3 ± 13.0	52.6 ± 12.3	47.9 ± 13.1	52.8 ± 15.3	58.9 ± 13.3	51.0 ± 15.5
BMI (kg/m^2^)	36.1 ± 7.9	35.8 ± 7.1	36.3 ± 8.3	30.3 ± 5.7	32.5 ± 7.2	29.7 ± 5.1
Diabetes	472 (46.9%)	182 (61.5%)	290 (40.8%)	58 (46.8%)	20 (69.0%)	38 (40.0%)
Steatosis grade						
1	330 (32.1%)	27 (9.1%)	303 (41.4%)	61 (49.2%)	12 (41.4%)	49 (51.6%)
2	355 (34.5%)	123 (41.4%)	232 (31.7%)	34 (27.4%)	9 (31.0%)	25 (26.3%)
3	344 (33.4%)	147 (49.5%)	197 (26.9%)	29 (23.4%)	8 (27.6%)	21 (22.1%)
Fibrosis stage						
0	196 (19.0%)	0 (0.0%)	196 (26.8%)	46 (37.1%)	0 (0.0%)	46 (48.4%)
1	401 (39.0%)	0 (0.0%)	401 (54.8%)	31 (25.0%)	0 (0.0%)	31 (32.6%)
2	195 (19.0%)	151 (50.8%)	44 (6.0%)	22 (17.7%)	14 (48.3%)	8 (8.4%)
3	178 (17.3%)	146 (49.2%)	32 (4.4%)	19 (15.3%)	15 (51.7%)	4 (4.2%)
4	59 (5.7%)	0 (0.0%)	59 (8.1%)	6 (4.8%)	0 (0.0%)	6 (6.3%)
MASH present	660 (64.1%)	297 (100.0%)	363 (49.6%)	49 (39.5%)	29 (100.0%)	20 (21.1%)
Platelets (10^9^/L)	247 [205, 295]	245 [197, 290]	248 [208, 297]	224 [194, 282]	199 [139, 225]	233 [208, 300]
≥140	972 (96.0%)	277 (95.2%)	695 (96.3%)	109 (87.9%)	21 (72.4%)	88 (92.6%)
AST (IU/L)						
Female	32 [24, 46]	40 [30, 59]	30 [23, 42]	54 [28, 80]	77 [63, 93]	42 [26, 78]
Male	38 [28, 55]	48 [35, 62]	34 [26, 49]	42 [29, 70]	73 [56, 113]	38 [27, 49]
ALT (IU/L)						
Female	35 [24, 55]	45 [31, 72]	33 [23, 49]	63 [26, 103]	70 [58, 106]	50 [25, 98]
Male	52 [36, 82]	64 [44, 96]	46 [31, 72]	72 [46, 104]	105 [83, 144]	65 [40, 94]
VCTE (kPa)	7.8 [5.6, 11.3]	9.3 [7.1, 13.6]	6.8 [5.1, 9.8]			
CAP (dB/m)	333 [302, 366]	350 [320, 376]	326 [294, 359]			
FAST score	0.43 [0.23, 0.65]	0.61 [0.44, 0.73]	0.35 [0.18, 0.58]			
≥0.67	238 (23.6%)	113 (38.6%)	125 (17.5%)			
FIB-4 index	1.04 [0.73, 1.61]	1.36 [0.87, 1.85]	0.97 [0.69, 1.43]			
Shear wave speed (m/s)				1.45 [1.29, 1.73]	1.83 [1.58, 2.02]	1.40 [1.27, 1.56]
Dispersion slope (m/s/kHz)				11.7 [9.8, 14.7]	14.4 [11.8, 16.6]	11.0 [9.6, 13.3]
Attenuation coefficient (dB/cm/MHz)				0.76 [0.68, 0.85]	0.77 [0.69, 0.82]	0.76 [0.68, 0.86]

Note that “histological indication” is based on the “at-risk MASH” definition in registrational trials, meaning F2/F3 and metabolic dysfunction associated steatohepatitis (MASH). Numbers are counts (percentage), mean ± standard deviation or median [interquartile range]. ALT, alanine aminotransferase; AST, aspartate aminotransferase; CAP, controlled attenuation parameter; FAST score, Fibroscan AST score; FIB-4, fibrosis-4; VCTE, vibration-controlled transient elastography.

Patients with a histological indication for therapy differed from the remainder in the distribution of steatosis grades and fibrosis stages. In addition, they were somewhat older and had higher liver enzyme levels. Differences regarding ultrasound parameters are discussed below.

### Comparison of histology and NITs for fibrosis

Fig. 2 shows the well-known association between VCTE or SWS and histologically determined fibrosis stages. However, regardless of the NIT cut-offs chosen, there are considerable discrepancies in the classification of fibrosis stages. Moreover, the histological definition for treatment indication includes MASH, and cases with this indication often extend below the lower cut-offs and above the upper ones.

**Fig. 2:**
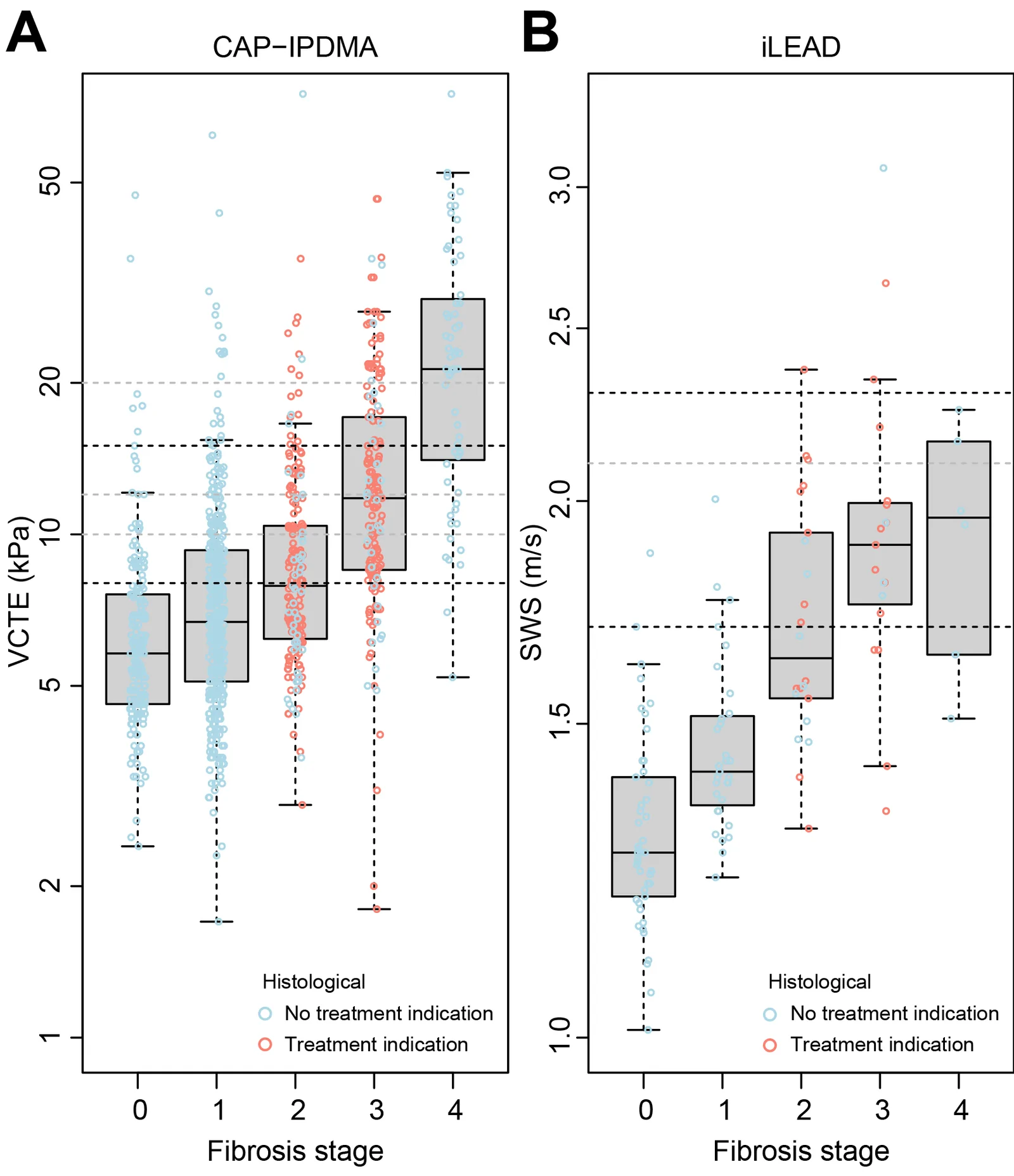
Liver stiffness measurements according to histologically determined fibrosis stage in CAP-IPDMA and iLEAD cohorts. (A) VCTE is shown as it depends on histologically determined fibrosis stage for CAP-IPDMA patients. The horizontal dashed lines at 8, 10, 12, 15 and 20 kPa are for orientation based on cut-offs from the expert recommendations. (B) SWS is shown as it depends on histologically determined fibrosis stage for iLEAD patients with cut-offs indicated at 1.7, 2.1 and 2.3 m/s. SWS, shear wave speed; VCTE, vibration-controlled transient elastography.

### Treatment indication according to histology and various NIT recommendations

Table 2 presents the number of patients who would qualify for treatment according to histological and NIT definitions. It also shows their characteristics.

**Table 2:** Patient characteristics based on different strategies to determine indication for therapy.

Platelets ≥140 × 10^9^/L:	CAP-IPDMA						iLEAD		
	Histology n = 277	8–15 kPa n = 329	10–15 kPa n = 173	10–20 kPa n = 215	12–20 kPa n = 136	FAST ≥0.67 n = 220	Histology n = 21	1.7–2.1 m/s n = 17	1.7–2.3 m/s n = 20
Number of females	131 (47.3%)	180 (54.7%)	98 (56.6%)	114 (53.0%)	65 (47.8%)	98 (44.5%)	11 (52.4%)	10 (58.8%)	11 (55.0%)
Age (years)	52.4 ± 12.5	50.2 ± 12.8	50.4 ± 13.0	51.3 ± 13.0	50.7 ± 13.0	50.2 ± 13.4	58.0 ± 12.7	54.1 ± 19.0	55.0 ± 17.7
BMI (kg/m^2^)	36.0 ± 7.2	36.0 ± 8.0	36.1 ± 7.7	36.2 ± 7.7	37.3 ± 7.7	37.6 ± 7.7	33.2 ± 6.6	32.3 ± 6.3	32.0 ± 5.9
Diabetes	173 (62.7%)	183 (57.0%)	102 (60.0%)	130 (61.3%)	85 (63.0%)	121 (55.5%)	15 (71.4%)	10 (58.8%)	11 (55.0%)
Steatosis grade									
1	25 (9.0%)	91 (27.7%)	47 (27.2%)	60 (27.9%)	37 (27.2%)	35 (15.9%)	6 (28.6%)	6 (35.3%)	7 (35.0%)
2	115 (41.5%)	124 (37.7%)	66 (38.2%)	79 (36.7%)	46 (33.8%)	63 (28.6%)	9 (42.9%)	5 (29.4%)	6 (30.0%)
3	137 (49.5%)	114 (34.7%)	60 (34.7%)	76 (35.3%)	53 (39.0%)	122 (55.5%)	6 (28.6%)	6 (35.3%)	7 (35.0%)
Fibrosis stage									
0	0 (0.0%)	34 (10.3%)	12 (6.9%)	16 (7.4%)	11 (8.1%)	6 (2.7%)	0 (0.0%)	2 (11.8%)	2 (10.0%)
1	0 (0.0%)	123 (37.4%)	59 (34.1%)	68 (31.6%)	38 (27.9%)	64 (29.1%)	0 (0.0%)	4 (23.5%)	4 (20.0%)
2	143 (51.6%)	75 (22.8%)	40 (23.1%)	47 (21.9%)	29 (21.3%)	47 (21.4%)	11 (52.4%)	5 (29.4%)	6 (30.0%)
3	134 (48.4%)	83 (25.2%)	52 (30.1%)	70 (32.6%)	47 (34.6%)	73 (33.2%)	10 (47.6%)	5 (29.4%)	6 (30.0%)
4	0 (0.0%)	14 (4.3%)	10 (5.8%)	14 (6.5%)	11 (8.1%)	30 (13.6%)	0 (0.0%)	1 (5.9%)	2 (10.0%)
MASH present	277 (100.0%)	233 (70.8%)	128 (74.0%)	157 (73.0%)	102 (75.0%)	193 (87.7%)	21 (100.0%)	11 (64.7%)	14 (70.0%)
Platelets (10^9^/L)	247 [203, 291]	254 [214, 303]	251 [207, 299]	247 [204, 298]	247 [212, 304]	238 [194, 285]	209 [197, 242]	232 [202, 262]	226 [191, 250]
AST (IU/L)									
Female	40 [29, 60]	35 [25, 52]	39 [26, 66]	36 [27, 58]	36 [26, 59]	66 [47, 87]	79 [66, 102]	68 [47, 85]	69 [53, 101]
Male	48 [35, 62]	40 [30, 57]	41 [32, 60]	42 [32, 66]	46 [33, 75]	59 [44, 84]	73 [53, 99]	71 [43, 99]	76 [44, 114]
ALT (IU/L)									
Female	47 [32, 73]	43 [25, 70]	45 [25, 72]	43 [25, 69]	39 [25, 57]	58 [43, 87]	78 [68, 114]	90 [36, 119]	78 [45, 114]
Male	63 [43, 96]	54 [37, 83]	57 [39, 81]	57 [40, 80]	56 [38, 82]	66 [47, 104]	96 [83, 144]	122 [96, 143]	122 [92, 146]

Only patients with platelets ≥140 × 10^9^/L are considered, since patients with lower values may have cirrhosis. Note that “Histology” is based on the “at-risk MASH” definition in registrational trials, meaning F2/F3 and MASH. The other column titles indicate which range of values are considered to imply indication for therapy, for example, 8-15 kPa means that patients with liver stiffness measurement in this range were considered to have the indication for therapy. Numbers are counts (percentage), mean ± standard deviation or median [interquartile range]. ALT, alanine aminotransferase; AST, aspartate aminotransferase; FAST, Fibroscan + aspartate aminotransferase; MASH, metabolic dysfunction-associated steatohepatitis.

For the CAP-IPDMA cohort, between 136 (13.2%) and 329 (32.0%) patients qualify for treatment according to the VCTE cut-off chosen. For the histological definition, 277 (26.9%) patients qualify. The prevalence of MASH ranges from 70.8% to 75.0% depending on the cut-offs for VCTE-based recommendations, but reaches 87.7% for the FAST score definition, which includes CAP and AST. Notably, different cut-offs result in different proportions of female patients, ranging from 44.5% to 56.6%.

For the iLEAD cohort, between 17 (13.7%) and 20 (16.1%) patients qualify for treatment according to the SWS cut-off chosen. For the histological definition, 21 (16.9%) patients qualify. The mean prevalence of MASH was 67% and 55% were female.

A graphical depiction of the overlap between the histological and NIT-based definitions can be found in Fig. 3. As an example, using the histological “at-risk MASH” definition and the VCTE 8-15 kPa definition, 132 (27.8%) of the 474 patients eligible for treatment according to at least one of the definitions fulfil both definitions. Using the histological “at-risk MASH” definition and the FAST ≥0.67 definition 107 (27.6%) of 387 patients fulfil both definitions. Adjustment of the VCTE cut-offs leads to less overlap and a smaller number of patients with an indication for therapy, *e.g*. at 12-20 kPa, 14% qualify for treatment and the overlap with the histological definition is 6%. The MPUS pilot data with the 1.7-2.3 m/s SWS cut-off are similar.

**Fig. 3:**
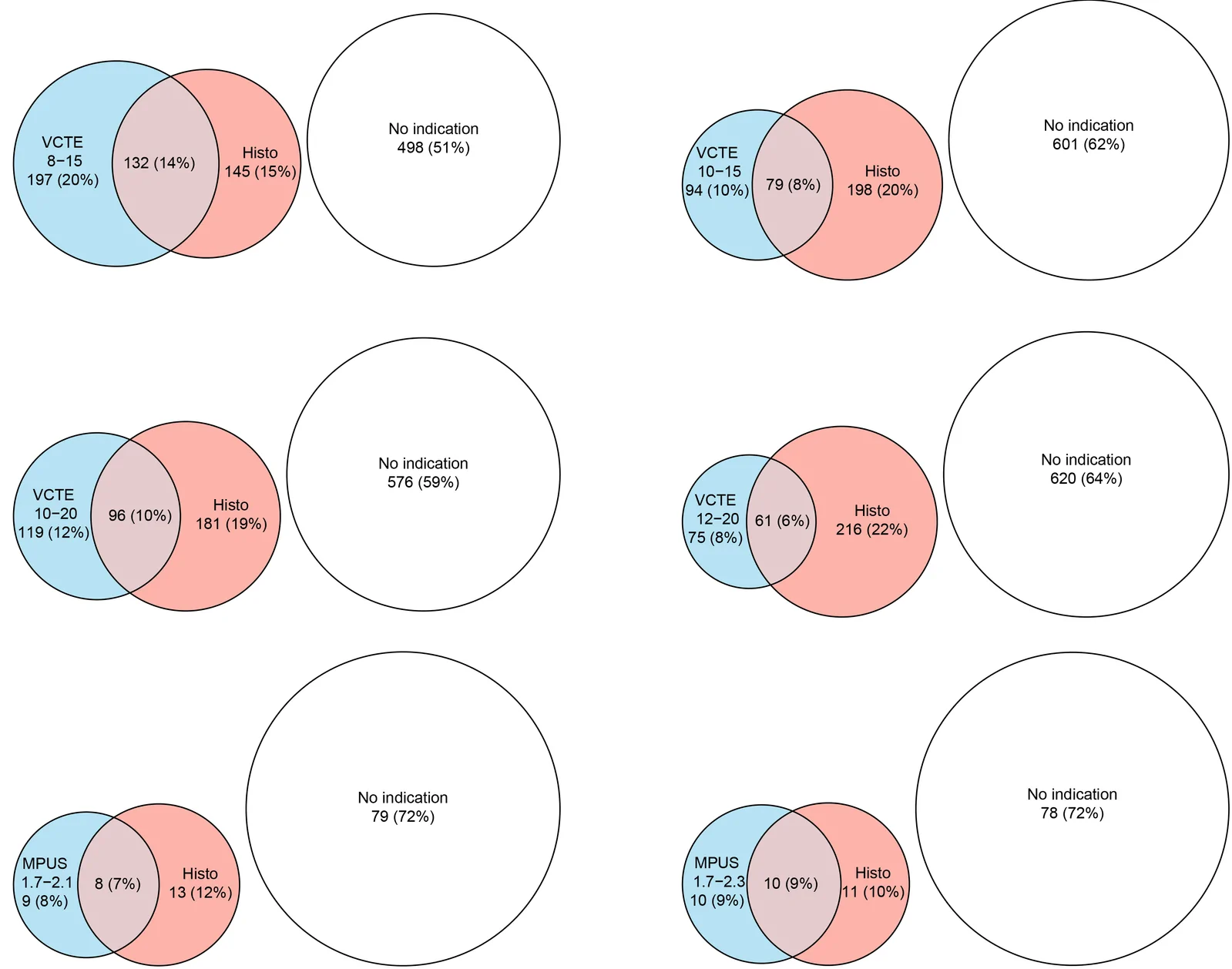
Overlap for treatment indication depending on the definition chosen. The proportion without treatment indication is also shown. The surface areas are proportional to the percentages. Note that here only the 972 CAP-IPDMA patients with platelet count ≥140 × 10^9^/L were included. MPUS, multiparametric ultrasound; VCTE, vibration-controlled transient elastography.

Taking the NIT-based definition of VCTE 8–15 kPa as an example, Table 3 compares patients according to different definitions of treatment eligibility. Patients with only this NIT indication are more likely to be female, are about 4 years younger on average, and are healthier in terms of diabetes, platelets, AST and alanine aminotransferase. Many of the patients with only the histological indication have low VCTE.

**Table 3:** Patient characteristics for those with therapy indication either according to histological criteria or based on an LSM of 8–15 kPa.

	Only histology n = 145	Only NIT n = 197	Both histology & NIT n = 132	*p* value (histology *vs*. NIT)	*p* value (histology *vs*. both)	*p* value (NIT *vs*. both)
Number of females	67 (46.2%)	116 (58.9%)	64 (48.5%)	**0.020**	0.70	0.063
Age (years)	51.9 ± 12.9	48.3 ± 13.0	52.9 ± 12.0	**0.012**	0.51	**0.0012**
BMI (kg/m^2^)	36.6 ± 6.8	36.5 ± 8.3	35.2 ± 7.6	0.96	0.13	0.16
Diabetes	88 (60.7%)	98 (51.6%)	85 (64.9%)	0.10	0.47	**0.018**
Steatosis grade				**<0.001**	0.89	**<0.001**
1	14 (9.7%)	80 (40.6%)	11 (8.3%)	–	–	–
2	61 (42.1%)	70 (35.5%)	54 (40.9%)	–	–	–
3	70 (48.3%)	47 (23.9%)	67 (50.8%)	–	–	–
Fibrosis stage					0.085	
0	0 (0.0%)	34 (17.3%)	0 (0.0%)	–	–	–
1	0 (0.0%)	123 (62.4%)	0 (0.0%)	–	–	–
2	82 (56.6%)	14 (7.1%)	61 (46.2%)	–	–	–
3	63 (43.4%)	12 (6.1%)	71 (53.8%)	–	–	–
4	0 (0.0%)	14 (7.1%)	0 (0.0%)	–	–	–
MASH present	145 (100.0%)	101 (51.3%)	132 (100.0%)	**<0.001**	–	**<0.001**
Platelets (10^9^/L)	245 [203, 287]	259 [214, 306]	251 [210, 299]	**0.037**	0.13	0.77
AST (IU/L)						
Female	37 [28, 52]	32 [23, 42]	47 [32, 64]	**0.034**	0.059	**<0.001**
Male	48 [32, 67]	35 [27, 55]	47 [36, 60]	**0.0072**	0.88	**0.0023**
ALT (IU/L)						
Female	42 [26, 64]	36 [23, 56]	52 [35, 83]	0.16	**0.035**	**<0.001**
Male	62 [41, 99]	45 [34, 66]	65 [44, 91]	**0.0016**	0.90	**<0.001**
VCTE (kPa)	7.2 [6.1, 18.4]	9.8 [8.9, 12.0]	10.4 [8.9, 12.4]	–	–	0.11
<5	12 (8.3%)	0 (0.0%)	0 (0.0%)	–	–	–
5–7.9	83 (57.2%)	0 (0.0%)	0 (0.0%)	–	–	–
8–8.9	0 (0.0%)	58 (29.4%)	36 (27.3%)	–	–	–
9–10.9	0 (0.0%)	78 (39.6%)	36 (27.3%)	–	–	–
11–15	0 (0.0%)	61 (31.0%)	60 (45.5%)	–	–	–
15.1–19.9	17 (11.7%)	0 (0.0%)	0 (0.0%)	–	–	–
20–29.9	28 (19.3%)	0 (0.0%)	0 (0.0%)	–	–	–
≥30	5 (3.4%)	0 (0.0%)	0 (0.0%)	–	–	–
CAP (dB/m)	342.8 ± 48.6	334.2 ± 45.6	346.2 ± 36.0	0.10	0.51	**0.0082**

Only patients with platelets ≥140 × 10^9^/L are considered. The patients are divided into the groups of those with therapy indication according to the histological, but not the NIT (8–15 kPa) criterion, those with the NIT but not the histological criterion and those with both. *p* values for a particular comparison are not presented if the parameter in question was prescribed by the therapy indication, *e.g.* for the comparison of fibrosis stage in “histology *vs*. NIT”, the histology indication must have F2/F3 by definition. Numbers are counts (percentage), mean ± standard deviation or median [interquartile range]. ALT, alanine aminotransferase; AST, aspartate aminotransferase; CAP, controlled attenuation parameter; NIT, non-invasive test; VCTE, vibration controlled transient elastography. Chi-quared tests without continuity correction were used for categorical variables, the Welch *t*-test for variables provided as mean and standard deviation and the Wilcoxon-Mann-Whitney test were used for variables provided as median and interquartile range. *p*-values <0.05 are given in bold font.

### Exploratory analysis of attenuation and dispersion slope within MPUS

To assess whether MPUS parameters beyond stiffness may provide complementary information for future treatment stratification, we analysed AC and DS in the iLEAD cohort. The AC value was not associated with histologically determined indication for therapy. For patients with an indication for therapy, the AC value was −0.1 dB/cm/MHz lower (95% CI −0.7 to 0.5, *p* = 0.73) than in those without an indication. DS was associated with indication for therapy: per doubling of the DS value, the odds ratio for an indication for therapy was 1.3 (95% CI 1.1 to 1.5, *p* <0.001). However, DS and SWS are highly correlated (r = 0.68, 95% CI 0.58 to 0.77), suggesting that DS may not add independent discriminatory value to a stiffness-based model in this cohort.

## Discussion

Patient selection in MASLD for treatment options such as resmetirom or semaglutide is shifting from histological assessment to NITs.[15], [23], [31], [32], [33] This impacts which patients are treated, how they are monitored and to what extent liver-related endpoints and overall mortality are finally reduced. Our study showed that there is little overlap between the patients selected, depending on which method and cut-offs are used. The total number of patients with an indication for treatment is roughly comparable using the histological “at-risk MASH” definition and the VCTE 8-15 kPa definition; however, only 28% of the patients potentially eligible fulfil both definitions. Adjustment of the VCTE cut-offs leads to even less overlap and reduces the number of patients with an indication for therapy. The 8-15 kPa definition includes earlier disease stages compared to the registrational trials.[9], [10] Higher cut-offs encompass cACLD, which goes beyond the trial definitions. Analysis of the MPUS pilot data using the 1.7–2.3 m/s cut-offs points in the same direction. The FAST score identifies the highest proportion of patients with histologically identified MASH, but the overlap with the histological definition is only 28%.

In the Research Letter by John *et al.*, 25/291 (9%) patients from the German MASLD Clinical Study Group with VCTE values between 10-20 kPa were eligible for treatment according to the histological criteria,^25^ which agrees with 96/942 (10%) from our study. Complete endorsement of non-invasive criteria would imply that 66/291 (23%) of patients from their study are eligible for treatment and 215/942 (23%) from our study. In 266 patients from the Turkish NAFLD Biobank, Kaya *et al.* found that 31% of patients with VCTE values between 10 and 20 kPa also fulfilled the histological definition for treatment.^26^ Because of strong preselection criteria (*e.g*. CAP ≥280 dB/m and no cirrhosis), it is difficult to compare numbers.

Several studies have demonstrated a high diagnostic accuracy of VCTE in predicting an increased risk of clinically relevant outcomes such as liver-related events, hepatic decompensation or HCC.[34], [35], [36], [37], [38] In particular, an increase in VCTE values over time has been shown to correlate with higher risk of hepatic complications in patients with chronic liver disease.[35], [39] Recently, Zhang *et al.* showed that VCTE and histological fibrosis staging are comparable for predicting hepatic events in patients with MASLD. This supports the paradigm shift toward NIT-based risk stratification and treatment decision-making in MASLD.^40^ Likewise, 2D-SWE has been identified as an independent predictor of mortality and hepatic decompensation.[21], [35], [41] Hence, these NITs can be considered stand-alone diagnostic and prognostic decision tools. In clinical practice, histology is hardly feasible for large patient numbers, while VCTE can be readily implemented. Among the currently available NITs, VCTE is the most widely used tool in everyday practice.^42^ However, emerging technologies such as MPUS incorporating 2D-SWE, AC, and DS offer promising alternatives. In the present analysis, however, patient selection for potential treatment eligibility was based on SWS cut-offs alone. Given that AC and DS have been proposed to reflect hepatic steatosis and inflammation,^22^ their combination with SWS may provide complementary information for risk stratification. This hypothesis should be addressed in future prospective studies with sufficient patient numbers.

Importantly, liver biopsy and VCTE do not necessarily cover the same patient cohorts. Table 3 demonstrates that patient characteristics differ depending on the indication criteria chosen. Importantly, the registrational MAESTRO-NASH trial^9^ pre-selected based on VCTE >8.5 kPa meaning that the “both histology and NIT“ column is more similar to those patients than the “only histology” column. Interestingly, many of the “only histology” patients have low VCTE despite elevated liver enzymes, which may be the result of selection effects: the indication for a liver biopsy may have been due to elevated enzymes.

Therefore, there is no clear rationale to consider biopsy to be the “gold standard”. Several analyses have pointed out that the histological diagnosis of MASH *vs*. MASLD can be affected by sampling error and interobserver variability among pathologists, leading to changes in grading and staging.[43], [44], [45] As a result, the differences seen between NITs and biopsy results may be due in part to variability in the biopsy itself. Moreover, the commonly used endpoint “MASH resolution without worsening of fibrosis” has been criticised as unreliable, since inflammatory activity can fluctuate and fibrosis does not progress in a strictly linear way.^46^ Therefore, biopsy-based reference data may not be precise enough to serve as a gold standard for validating NITs.^47^ These considerations suggest that the focus should shift from the agreement between NITs and biopsy toward the ability of NITs to predict relevant clinical outcomes. It is now crucial to validate the clinical utility of NITs and the treatment recommendations derived from them in further analyses. In doing so, it will be necessary to decide clinically whether patients with more advanced disease (*e.g*. VCTE 12-20 kPa) are considered eligible for treatment, if wider cut-offs are chosen to include a broader population (*e.g.* VCTE 8-15 kPa) or if MASH is retained as a central criterion (*e.g*. FAST ≥0.67). For the time being, clinicians should be aware that VCTE measurements >7.9 kPa are not reproducible in one third of cases with MASLD upon serial measurements.^48^ Therefore, the indication for MASH treatment may not be based on single evaluations, but rather on follow-up VCTE results.^25^ In cases with persistently elevated VCTE, it is difficult to assess to what extent these patients are similar to “only NIT” or “both”, but outcome signals for these groups of patients suggest that treatment may be justified.^49^ A critical appraisal of patients with a histological indication for therapy but low VCTE is warranted, since such patients are hardly represented in trials to date. Ongoing open-label extension studies are evaluating the safety and tolerability of resmetirom using primarily NIT-based monitoring (ClinicalTrials.gov, IDs: NCT05500222 and NCT04951219). In addition, a dedicated NIT-based outcome study has recently been initiated (ClinicalTrials.gov, ID: NCT07165028), further supporting the evaluation of treatment response and clinical outcomes using non-invasive assessment strategies. For widespread use in real-world scenarios, future diagnostic approaches should aim to be readily applicable in routine clinical practice rather than being confined to tertiary care centres.

Our data are based on a large number of biopsy-proven individual data originating from various international clinical projects. However, differences in the distribution of biopsy results between the iLEAD cohort and the CAP-IPDMA group indicate selection bias, because individuals in the two cohorts may have undergone liver biopsy for different reasons and because histology readings were central in the iLEAD cohort.

The most prominent limitation of this study is that we performed a secondary analysis of data which were not collected primarily to answer the question addressed. However, they are derived from clinical scenarios where pharmacological treatment is considered. A further limitation is that VCTE was not performed in the iLEAD cohort, meaning that a direct comparison of MPUS and VCTE is not possible. In addition, the data in the iLEAD cohort were obtained from a single ultrasound vendor, which may limit the generalisability of the findings. Vendor-independent validation studies will be required to confirm the robustness of SWS-based cut-offs and their applicability across different MPUS platforms.

In conclusion, our analysis demonstrates that overlap in patients selected for treatment using histology-based criteria *vs*. NITs is small. The choice of cut-offs determines if a broader patient spectrum is considered for treatment or not. This analysis highlights the urgent need to further study NITs for patient selection and assessment, with the ultimate goal of establishing them as a reference standard commensurate with biopsy. Importantly, future clinical trials should be designed in closer alignment with real-world clinical practice, using the same non-invasive tools for both patient selection at trial entry and outcome assessment. Rather than relying on histology-based inclusion criteria and endpoints while NITs are applied in routine care, trials would ideally adopt NIT-based patient selection and NIT-derived reasonably likely surrogate endpoints, thereby enhancing the clinical applicability, external validity and translational relevance of trial results.

## Abbreviations

2D-SWE, two-dimensional shear wave elastography; AC, attenuation coefficient; AST, aspartate transaminase; cACLD, compensated advanced chronic liver disease; CAP, controlled attenuation parameter; DS, dispersion slope; FAST, Fibroscan + aspartate aminotransferase; FIB-4, fibrosis-4; HCC, hepatocellular carcinoma; LSM, liver stiffness measurement; MASH, Metabolic dysfunction-associated steatohepatitis; MASLD, Metabolic dysfunction-associated steatotic liver disease; MPUS, multiparametric ultrasound; NAS, NAFLD Activity Score; NITs, non-invasive tests; SWS, shear-wave speed; VCTE, vibration-controlled transient elastography.

## Authors’ contributions

Conceptualization: TK, JW, DP. Methodology: TK, JW, DP. Data acquisition: all authors. Formal analysis: TK, JW, DP, EKM. Writing - original draft preparation: TK, JW, DP, EKM. Writing - review & editing: all authors.

## Data availability

For data availability, please contact the corresponding authors of the original papers.

## Financial support

The study was funded by the 10.13039/501100001659Deutsche Forschungsgemeinschaft (DFG, 10.13039/501100001659German Research Foundation) under Germany´s Excellence Strategy – EXC-3105/1 – 533765739.

## Conflicts of interest

EKM: Travel support by Mirum Pharmaceuticals. MDB: Payment for presentation by Canon Medical Systems. VB: Unrestricted research funding from Mindray and Canon Medical Systems outside the submitted work. Travel support was provided by Canon Medical Systems (Study Meeting iLEAD and lectures) and Mindray (lectures). The author received lecture honoraria from Mindray, Canon Medical Systems, Boehringer Ingelheim Pharma, and the Falk Foundation e.V. WKC: A consultant or advisory board member for Abbott, Abbvie, Boehringer Ingelheim, IPSEN, Kowa, Novo Nordisk, Roche, and Zuellig Pharma, a speaker for Abbott, Echosens, Hisky Medical, Novo Nordisk, Roche and Viatris, and receiving research grants from Abbott and Roche. VC: Payment or honoraria for lectures, presentations, speakers bureaus, manuscript writing, or educational events from Bracco, Canon, Siemens, Samsung, and Mindray; support for attending meetings and/or travel from Bracco, Esaote, Samsung, Siemens, Canon, and Mindray; president elect of the European Federation for Ultrasound in Medicine and Biology; past president of the Società Italiana di Ultrasonologia in Medicina e Biologia; and US section chairman of the Italian Society of Medical and Interventional Radiology. GF: Grants or contracts to institutions from Canon Medical Systems and Esaote; royalties or licenses from Elsevier; payment or honoraria for lectures, presentations, speakers bureaus, manuscript writing, or educational events from Canon Medical Systems, Fujifilm, Mindray Medical Imaging, and Philips Ultrasound; participation on a data and safety monitoring board or advisory board for Philips Healthcare; and equipment received by the institution from Esaote and Fujifilm. Secretary of the World Federation for Ultrasound in Medicine and Biology and Chair of the Publication Committee. EGG: Multicentre grant to institution from Canon Medical Systems and grants or contracts from Siemens Healthineers; consultant for Radiology. HG: Member of the board of the Society of Radiologists in Ultrasound and chair of the American College of Radiology US Guidelines Committee. JG: Clinical research funding and US scanner loan to institution to support this study from Canon Medical Systems; principal investigator on this study funded by Canon Medical Systems; honorarium for lecture from RSNA. HI: Research grants from Canon Medical Systems and GE HealthCare. TK: Financial support to the institution for conducting the ILEAD study, provision of software for the US system, and travel expenses for study meetings from Canon Medical Systems; provision of material (FibroScan) for clinical studies on related topics from Echosens; payment or honoraria for lectures and speaking from Jazz Pharmaceuticals and Echosens; participation on a clinical advisory board for Echosens; speaker of the Sonography Working Group of the German Society for Gastroenterology, Digestive and Metabolic Diseases; head of the Internal Medicine Section of the German Society of Ultrasound in Medicine. AL: Payment for speakers bureau and loan of equipment from Canon Medical Systems. VdL: became an employee of Echosens after collecting the data used in this manuscript, but before the analyses were performed and the manuscript drafted. DHL: Research grant from Canon Medical Systems. JYL: Korean national grants for the development of histotripsy and brain device; Focused Ultrasound Foundation grants for clinical research of high-intensity focused ultrasound; vice president of the Asian Federation of Societies for Ultrasound in Medicine and Biology; board member of the International Society for Therapeutic Ultrasound; stock options from the International Immunogenetics Information System. FM: Institution has a research contract from Canon Medical Systems to evaluate a new function of the Canon US machine; institution receives grants and rents US equipment from Canon Medical Systems; director of Medilec, which has a consulting contract and receives honoraria for consulting on this study, workshops, conference presentations, manuscript submission and publishing; travel expenses from Canon Medical Systems. DP: receipt of unrestricted research grants from Echosens. VW: Consultant or advisory board member for AbbVie, AstraZeneca, Boehringer Ingelheim, Echosens, Eli Lilly, Gilead Sciences, Merck, Novo Nordisk, Pfizer, Roche Diagnostics, TARGET PharmaSolutions; speaker for Abbott, AbbVie, AstraZeneca, Echosens, Gilead Sciences, Novo Nordisk; research grant from Gilead Sciences; co-founder of Illuminatio Medical Technology. JW: lecturer and advisory board member for Abbvie, Intercept/Advanz Pharma, GSK, Ipsen, Gilead Sciences, NovoNordisk, Madrigal; receipt of grants/research supports from Abbvie, Gilead Sciences, NovoNordisk, Echosens. The remaining authors have nothing to disclose.

Please refer to the accompanying ICMJE disclosure forms for further details.
